# Rhus Toxicodendron

**Published:** 1891-03-21

**Authors:** 


### THERAPEUTICS.

### RHUS TOXICODENDRON.

Many years ago Dr. Wood, of Philadelphia, made some therapeutical investigations with this drug, but was not able to obtain any uniform or definite results. His attention was chiefly directed to cases of chronic rheumatism. The drug has, however, never come into general use, in fact, the majority of practitioners have probably never heard of it by name even. In a recent communication to the *Therapeutic Gazette*, Dr. John Aulde tells us that some years ago, while looking up a desirable remedy for the relief of an obstinate case of sciatic rheumatism, his attention was attracted by some remarks dropped by one of his patients, who claimed that he had long suffered from what is termed varicose veins and from chronic rheumatism, which the doctors had pronounced incurable. He was advised to obtain a preparation made from the poison sumach, which is referred to generally as rhus toxicodendron. He (the patient) told him that he was advised only to take about five drops, but that he found by experience that he could take as much as nine drops without feeling any bad effects. With a view to obviate any dangerous symptoms that might arise from its use, he began with very small doses, and we believe that herein lies the success Dr. Aulde obtained from its use, and which, he tells us, so agreeably surprised him. The preparation that he uses is the tincture prepared according to the directions of the Pharmacopœia for the making of tinctures from fresh herbs —fifty parts of the fresh leaves to one hundred parts of alcohol. The dose of this tincture is half a drop, and thus, for convenience of dispensing, it is prepared with diluted alcohol—one part of the tincture to ten of the diluted alcohol; the dose of this preparation being, of course, five drops. This certainly will be found the most suitable method of administering the drug.

There are about eighty varieties of the rhus family, and most of these exhibit poisonous properties. They give off, when cut, acrid vapour which is intensely irritating. When absorbed, or internally administered, rhus products give rise to an intense irritation of the skin, producing an eczematous condition. Moderately large doses result in vomiting and diarrhœa. The connective tissues of the muscles and their tendons have been found much inflamed in rhus poisoning. It would seem that just as strychnine exerts its peculiar effects on certain portions only of the spinal cord, so rhus picks out the connective tissue, and the skin, and exerts its peculiarly stimulating properties on them. In the article we have referred to above, Dr. Aulde quotes several cases reported to him by various practitioners who have used this remedy, and they illustrate so admirably the kind of case in which rhus may be used that we will quote one of them verbatim. This case was that of a "male aged thirty-eight, who was attacked with a severe pain, most certainly in the sciatic nerve; had pain constantly for four weeks. Was treated by three other physicians. Was treated with everything in the way of medicine known to the practitioner, all to no purpose. Was becoming weak and nervous for want of rest and food. He was advised to go to Chicago, and he did so, remaining in one of the hospitals, attended by one of Chicago's best physicians for ten days, when he came home about as he went. He intended to return to Chicago in a few days. The reporter saw him, and asked him to try another remedy. Gave the tincture of rhus toxicodendron diluted, with directions to take five drops in water three times daily. After taking five doses he awoke in the morning scarcely able to find his sciatic pain. Felt only a few twitches of it that day, and has never felt it since; feels well and strong." Many other equally favourable reports are given by Dr. Aulde. In one case of hæmorrhoids the reporting physician was himself the patient, and he was greatly relieved by taking the rhus. On leaving off the rhus the hæmorrhoids soon became as bad as before, but on again resuming the medicine they became greatly improved. In another case cystitis was benefited.

Our own experience with the drug has been almost confined to skin diseases, and for certain cases of eczema it has done wonders. It is difficult to indicate what cases of eczema may be regarded as most likely to be benefited by rhus, but speaking generally we would say that they are those cases which do not appear to have any attributable cause, but which seem to be due to a diathetic weakness, and probably result from the condition of the trophic nerves of the affected parts. In eczema due to local irritation, or to local congestion in association with varicose veins of the leg, for instance, or in cases where it is obviously due to constipation or other intestinal troubles, we have already very much more efficient therapeutical measures adapted to the various cases. It is rather in the chronic eczemas that seem to resist all our efforts to cure that rhus may be found to give most happy results.

